# Gunshot Wounds of the Chest

**Published:** 1918-10

**Authors:** 


**Some Features of Gunshot Wounds of the Chest.** By J. F. Dobson, Lieutenant-Colonel, R. A. M. C. Abs. from the *British Medical Journal,* June 15, 1918.

The chief cause of failure in operations on the chest is sepsis. The writer describes two cases in which chest wounds were excised and sutured, and the patients evacuated. In both, serious infection, necessitating opening and drainage, set in. The writer, therefore, considers it dangerous to evacuate a case in which the chest has been primarily sutured, until the possibility of infection is past. According to Gask and Wilkinson[1], such infection after closure occurs in about 28 per cent. of cases. Anderson[2] reports 24 per cent., and Roberts and Graig[3] 40 per cent., of such infection.

Although early and thorough operative treatment with closure of the chest is the best preventive of sepsis, there are many cases in which sepsis cannot be avoided, as, for instance, when it takes place in the lung itself. It is doubtful that treatment by drainage from the start would improve the results in these cases. Many succumb to sepsis in spite of large opening and free drainage. An infected chest wound must be treated, like all others, by washing the whole infected area at regular intervals with an antiseptic fluid, and by efficient drainage.

If symptoms indicating infection occur, the chest should be opened and explored and the hemothorax tested. Radiography is of much assistance in determining the amount of fluid, but it should not be allowed to delay operation. Clinical symptoms of infection should be regarded as sufficient indication for operation.

The operation may be performed in one of two ways :

1. Resection of a short section of a rib and the insertion of a drainage tube under local anesthesia ; or

1. *Brit. Med. Jour.* Dec. 15, 1917.
2. *Brit. Med. Jour.* Nov. 3, 1917.
3. *Brit. Med. Jour.* Nov. 3, 1917.

2. Thoracotomy under general or local anesthesia. Resection of a considerable length of rib (4 1/2 to 5 inches) preferably the 8th. Insertion of a spreader (Tuffier's *écarteur* is very satisfactory). Removal of clots, fluid, foreign bodies, bone fragments, etc., and repair of damage to lung and diaphragm.

Although the first operative treatment suffices in a majority of cases, drainage alone is not sufficient when sepsis is well established. A further disadvantage of the first procedure is that it does not permit of thorough exploration and cleansing.

Drainage should be supplemented by the instillation of an antiseptic. This may be accomplished by :

1. Irrigation of the cavity once or twice a day through a drainage tube. This method is effective only for a small cavity.

2. Irrigation every two hours through Carrel tubes supported on wires and inserted into the cavity in all directions through the drainage aperture. (Tuffier's method.)

3. Filling of the cavity with Dakin's solution every four hours through the drainage tube. The fluid should be siphoned off at the end of two hours.

4. Irrigation through a special silver cannula[1]. This is sutured into the third or fourth space and a drainage tube is sutured into the original incision. A Carrel ampoule is then attached by a connecting tube to the cannula. Thus the cavity is irrigated every two hours.

The author points out many advantages to be derived from this last method of procedure. The results from it have been striking. The patient's condition has improved rapidly and the lung has expanded. If the cannula has been removed because of pain from the lung expansion, an ordinary Carrel tube may replace it.

Various methods of treating chronic empyema are discussed. The author objects to methods which, like Estlander's, Schnede's, and Wilms's, merely fill the cavity and do not permit of the expansion of the lung. Decortication of the lung after an osteoplastic resection, by which procedure the thickened visceral pleura is stripped from the surface of the lung exposed in the cavity, sometimes induces expansion, but is dangerous and not to be recommended.

In two forms of empyema, in which the collapse of the lung is not complete, the lung is prevented from expanding by a fibrinous growth of membrane which attaches more or less rigidly the pleural membrane covering of the lung to the lining of the chest wall.

1. *Brit. Med. Jour.*, Feb. 2, 1918, for description of the cannula by the author.

This attachment is especially strong at the two points where the lung remains in contact with the chest wall (See Fig. A and F). If this membrane has already formed, as is the case after any delay in recognizing and draining the empyema, it effectively prevents complete lung expansion. If these adhesions can be separated, expansion is assured. The operation is not difficult and there is little danger from hemorrhage.

When, however, the collapse of the lung is complete, the cavity can be closed only by an extensive osteoplastic operation.

